# Functional interaction of Rpb1 and Spt5 C-terminal domains in co-transcriptional histone modification

**DOI:** 10.1093/nar/gkv837

**Published:** 2015-08-14

**Authors:** Jean Mbogning, Viviane Pagé, Jillian Burston, Emily Schwenger, Robert P. Fisher, Beate Schwer, Stewart Shuman, Jason C. Tanny

**Affiliations:** 1 Department of Pharmacology and Therapeutics, McGill University, Montreal, Quebec, H3G 1Y6, Canada; 2 Department of Oncological Sciences, Icahn School of Medicine at Mount Sinai, New York, NY 10029, USA; 3 Department of Microbiology and Immunology, Weill Cornell Medical College, New York, NY 10065, USA; 4 Molecular Biology Program, Memorial Sloan-Kettering Cancer Center, New York, NY 10065, USA

## Abstract

Transcription by RNA polymerase II (RNAPII) is accompanied by a conserved pattern of histone modifications that plays important roles in regulating gene expression. The establishment of this pattern requires phosphorylation of both Rpb1 (the largest RNAPII subunit) and the elongation factor Spt5 on their respective C-terminal domains (CTDs). Here we interrogated the roles of individual Rpb1 and Spt5 CTD phospho-sites in directing co-transcriptional histone modifications in the fission yeast *Schizosaccharomyces pombe*. Steady-state levels of methylation at histone H3 lysines 4 (H3K4me) and 36 (H3K36me) were sensitive to multiple mutations of the Rpb1 CTD repeat motif (Y_1_S_2_P_3_T_4_S_5_P_6_S_7_). Ablation of the Spt5 CTD phospho-site Thr1 reduced H3K4me levels but had minimal effects on H3K36me. Nonetheless, Spt5 CTD mutations potentiated the effects of Rpb1 CTD mutations on H3K36me, suggesting overlapping functions. Phosphorylation of Rpb1 Ser2 by the Cdk12 orthologue Lsk1 positively regulated H3K36me but negatively regulated H3K4me. H3K36me and histone H2B monoubiquitylation required Rpb1 Ser5 but were maintained upon inactivation of Mcs6/Cdk7, the major kinase for Rpb1 Ser5 *in vivo*, implicating another Ser5 kinase in these regulatory pathways. Our results elaborate the CTD ‘code’ for co-transcriptional histone modifications.

## INTRODUCTION

The RNA polymerase II (RNAPII) elongation complex coordinates transcription with key co-transcriptional events such as mRNA processing and histone post-translational modification. Association of processing factors and regulatory partners with the RNAPII elongation complex generally depends upon Rpb1, the RNAPII large subunit, and/or Spt5. Rpb1 and Spt5 have distinctive C-terminal domains (CTDs) comprising multiple repeats of a short motif; their CTDs interact with multiple regulators and are critical for assembly of a functional elongation complex (1,2). Whereas the canonical heptapeptide Rpb1 CTD motif (Y_1_S_2_P_3_T_4_S_5_P_6_S_7_) is conserved across diverse eukaryotic species, the number of repeats in the array increases with progression up the evolutionary scale. In contrast, the Spt5 CTD repeat array is variable, between taxa and within the Spt5 CTD array of a given species. The consensus repeated motif in human Spt5 is TP(M/L)YGS(R/Q) (3,4). The fission yeast *Schizosaccharomyces pombe* Spt5 contains a relatively homogeneous CTD consisting of 19 tandem copies of consensus TPAWNSGSK. The consensus repeat in budding yeast Spt5 (referred to as the CTR) is highly divergent (SAWGGQ) (5–7). Multiple components of the RNAPII elongation complex can bind to both CTD domains, and genetic analysis indicates that the two CTDs have overlapping functions *in vivo* (1,2,5,8–10).

The Rpb1 and Spt5 CTDs direct co-transcriptional histone modifications, which are present in a stereotyped pattern within the coding regions of transcribed genes. Signature features of this program are a 5′ peak of trimethyl H3K4 and a 3′ peak of trimethyl H3K36. Mono-ubiquitylated histone H2B (H2Bub1), methylated H3K79 and dimethyl forms of both H3K4 and H3K36 are all found broadly distributed across coding regions. These modifications have roles in nucleosome dynamics, mRNA processing and DNA repair (11–16). How the activities of the relevant histone-modifying enzymes are coordinated with the RNAPII elongation complex to generate this pattern is an important unresolved question (17).

Underlying the program of co-transcriptional histone modifications is an equally complex pattern of phosphorylation on the Rpb1 and Spt5 CTDs, which regulates their interactions with elongation factors. All five of the potential phosphoacceptor sites within the Rpb1 CTD motif are phosphorylated at various stages of the transcription cycle (18). Most phospho-specific interactions with the Rpb1 CTD studied to date involve the phospho-Ser2 and phospho-Ser5 isoforms (Rpb1 S2-P and Rpb1 S5-P). Rpb1 S5-P and Rpb1 S2-P show characteristic distribution patterns within RNAPII transcription units: Rpb1 S5-P is preferentially enriched near promoters, whereas Rpb1 S2-P peaks toward gene 3′ ends. Numerous biochemical and structural studies of phospho-CTD interaction domains recognizing Rpb1 S2-P and Rpb1 S5-P have reinforced the notion that Rpb1 S5-P acts early in elongation, whereas Rpb1 S2-P acts later. For example, mRNA capping enzymes, which act on nascent mRNA 5′ ends, are directly engaged and allosterically activated by Rpb1 S5-P (8,19). On the other hand, factors involved in mRNA 3′ end cleavage and polyadenylation directly bind to Rpb1 S2-P (2,20). The Spt5 CTD is typically phosphorylated on threonine of the repeat sequence (the variant Spt5 CTD in budding yeast is phosphorylated on serine)(4,6,7,21,22). Functional studies indicate that this modification (Spt5 T1-P) positively influences elongation (4,5,7). Fission yeast capping enzymes bind to the unphosphorylated Spt5 CTD; this interaction is antagonized by threonine phosphorylation (8,9).

Rpb1 S5-P, Rpb1 S2-P and Spt5 T1-P have all been implicated in co-transcriptional histone modification. Biochemical experiments have demonstrated direct interactions between the Set1C/COMPASS H3K4 methyltransferase complex and Rpb1 S5-P, whereas the H3K36 methyltransferase Set2 interacts with Rpb1 CTD peptides phosphorylated on both Ser2 and Ser5 (23–25). The PAF complex, a multi-functional elongation factor that promotes H2Bub1, H3K4me and H3K36me, interacts with diphosphorylated Rpb1 S2-P/S5-P peptides as well as with Spt5 T1-P. These interactions involve the PAF subunits Ctr9 and Cdc73 (26,27). Rtf1, a protein that is functionally related to PAF and that is required for H2Bub1 and H3K4me, preferentially recognizes Spt5 T1-P (3,27–29). The human RNF20/RNF40/WAC complex, the E3 ubiquitin ligase for H2Bub1, preferentially associates with Rpb1 S2-P *in vitro* (30).

The *in vivo* approach to validate these interactions has been largely limited to genetic or chemical genetic inactivation of CTD kinases that target multiple sites. Cdk7, a major kinase for Ser5 and Ser7 on the Rpb1 CTD, promotes H2Bub1 and H3K36me in human cells (31,32). A temperature-sensitive variant of the *Saccharomyces cerevisiae* Cdk7 orthologue Kin28 also affects H2Bub1, whereas chemical genetic inhibition of the *S. pombe* orthologue Mcs6 only affects H3K4me (22,33). Cdk9, which phosphorylates the Spt5 CTD and multiple sites on the Rpb1 CTD, is implicated in H2Bub1, H3K4me and H3K36me (22,34–39). The *S. cerevisiae* Cdk12 orthologue Ctk1, a major kinase for Rpb1 Ser2, positively regulates H3K36me and negatively regulates H3K4me (40–42). H3K36me depends on Cdk12 in the nematode *Caenorhabditis elegans* as well (43). While these studies have highlighted the importance of some combination of Rpb1 S5-P, Rpb1 S2-P and Spt5 T1-P for co-transcriptional histone modifications, they do not address the contributions of individual sites and how the sites functionally interact. The potential roles of other Rpb1 CTD phosphorylation sites in establishing the co-transcriptional histone modification pattern have not been assessed.

A more detailed understanding of the roles of individual residues in the Rpb1 and Spt5 CTD repeats is beginning to emerge from mutagenesis studies conducted in a variety of model systems (5,44–46). Here we systematically profile multiple co-transcriptional histone modifications across a series of Rpb1 and Spt5 CTD mutants, as well as in mutants affecting CTD kinases. Our findings provide novel insights into the mechanisms linking the histone and CTD modification programs.

## MATERIALS AND METHODS

### *pombe* strains and media

Strains harboring mutations at the *rpb1*^+^ and *spt5*^+^ loci, as well as analogue-sensitive alleles of *mcs6*^+^, *cdk9*^+^ and *lsk1*^+^, have been described previously (44,47,48). The *spt5-CTD-7 rpb1-S7A, spt5-T1A rpb1-S7A, cdk9^as^ lsk1^as^* and *mcs6^as^ rpb1* strains were generated using genetic crosses and tetrad analysis (49). The relevant allele combinations were confirmed using diagnostic polymerase chain reaction (PCR) and western blotting. The ‘WT’ (wild-type) strain used in Figures 2–4, Supplementary Figures S1, S2 and S4 is strain JS78 and has been described previously (22). The *cdk9-T212A, set1*Δ and *htb1-K119R* strains have been described previously (22,50). The *set2*Δ and *set9*Δ strains were constructed by replacement of the *set2*^+^ or *set9*^+^ coding regions with a hygromycin resistance cassette as described previously (51). The *lsk1^as^* allele was amplified by PCR and introduced at its native locus in the *rpb1-CTD-14* or the *rpb1-S2A* strain to create the combined *lsk1^as^ rpb1* mutants. The *lsk1*Δ strain was generously provided by Jim Karagiannis (52).

Liquid cultures were grown at 30°C using standard YES media (yeast extract 5 g/l, D-glucose 30 g/l, supplemented with 250 mg/l each of histidine, leucine, adenine and uracil). 3-MB-PP1 (purchased from Toronto Research Chemicals) was dissolved in dimethyl sulfoxide (DMSO) at a final concentration of 50 mM. For inhibition of analogue sensitive alleles, cells were grown to early log phase (OD_600_ 0.2) and treated with the indicated concentration of 3-MB-PP1 for 3 h. Mycophenolic acid (MPA; purchased from Bioshop Inc.) was dissolved in DMSO and added to solid YES media at a concentration of 25 μg/ml.

### Immunoblotting

Preparation of *S. pombe* whole-cell extracts, SDS-PAGE, and immunoblotting were performed as previously described (22). The following commercial antibodies were used: H2Bub1 (Active Motif #39623), H3K4me1 (Abcam #ab8895), H3K4me2 (Abcam #ab32356), H3K4me3 (Abcam #ab8580), H3K36me3 (Abcam #ab9050), histone H3 (Abcam #ab1791), Rpb1 (8WG16; Covance #MMS-126R-200), Rpb1 S2-P (clone 3E10; Millipore #04–1571), Rpb1 S5-P (clone 3E8; Millipore #04–1572). Antibodies against *S. pombe* Spt5 and Spt5 T1-P were described previously (22).

### Chromatin immunoprecipitation (ChIP)

ChIP was carried out as previously described (28). The Spt5 antibody recognizing the CTD was used at a concentration of 5 μg/ml of extract. A strain lacking the entire CTD (*spt5*Δ*CTD*) was used as a negative control.

## RESULTS

### Comprehensive analysis of the roles of Rpb1 CTD phosphorylation sites in co-transcriptional histone modification

**Figure 1.**
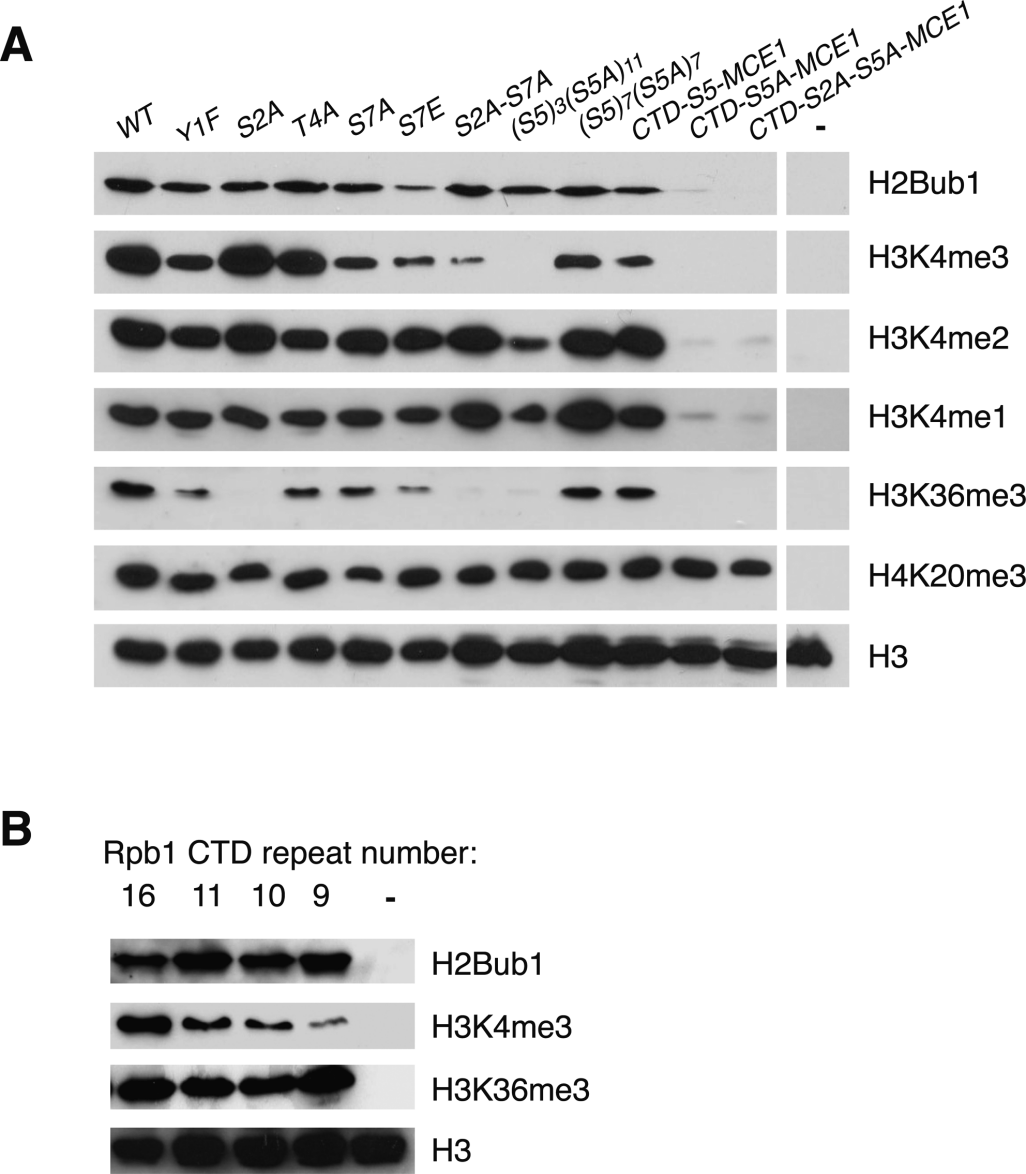
Analysis of the roles of Rpb1 CTD phosphorylation sites in co-transcriptional histone modifications. **(A)** Whole-cell extracts derived from the indicated *rpb1* strains (top) were subjected to SDS-PAGE and immunoblotting with the indicated antibodies (right). The ‘WT’ strain expresses a truncated but fully functional form of the CTD containing 14 consensus heptapeptide repeats (*rpb1-CTD-14*). The mutant strains express CTDs bearing the indicated mutations in all of the consensus repeats. The *S5A* mutant strains harbor CTDs with the indicated combinations of wild-type and *S5A* mutant repeats. MCE1 strains express CTDs that are fused to the mRNA capping enzyme MCE1. The last lane on each blot shows an extract derived from a negative control strain (*htb1-K119R* for H2Bub1, *set1*Δ for H3K4me, *set2*Δ for H3K36me3, *set9*Δ for H4K20me3). **(B)** As in (A). Shown are immunoblots performed on extracts from strains expressing *rpb1* variants with 16, 11, 10 or 9 consensus repeats. The last lane on each blot shows an extract derived from a negative control strain (*htb1-K119R* for H2Bub1, *set1*Δ for H3K4me3, *set2*Δ for H3K36me3).

Our analysis employed a series of Rpb1 mutants in the fission yeast *S. pombe* in which a truncated but fully functional CTD (consisting of 14 consensus heptad repeats and the four non-consensus ‘rump’ repeats proximal to the body of Rpb1) was replaced with CTD variants harboring single amino acid substitutions in all or some of the repeats (44). To assess the role of the essential Ser5 position, we used either strains in which varying combinations of Ser5-Ala (S5A) repeats and wild-type Ser5 repeats were present, or strains in which lethality of complete S5A substitution of all consensus heptads was bypassed via Rpb1 fusion to the mammalian mRNA capping enzyme MCE1 (44,47). Steady-state levels of H2Bub1, H3K4me (H3K4me1, H3K4me2 and H3K4me3), and H3K36me3 were evaluated by immunoblotting of whole-cell extracts. As controls, we also immunoblotted total histone H3 and histone H4 lysine 20 methylation (H4K20me), a modification with no known connection to transcription (53). Controls for antibody specificity included the following mutant strains: *htb1-K119R* (lacking H2Bub1; (54)), *set1Δ* (lacking H3K4me; (55)), *set2Δ* (lacking H3K36me; (56)) and *set9Δ* (lacking H4K20me; (53))(Figure 1A).

We found that S5A mutation dramatically reduced the levels of all the co-transcriptional histone modifications tested without affecting H4K20me, affirming the general importance of this site for coupling histone modifiers to elongating RNAPII (Figure 1A). H3K4me1, H3K4me2, H3K4me3 and H3K36me3 were reduced in the (S5)_3_(S5A)_11_ strain in which 11 of 14 Rpb1 CTD repeats were S5A, or a strain in which all 14 repeats were S5A in the context of an *rpb1-CTD-S5A-MCE1* translational fusion. However, there was no effect on these modifications in the (S5)_7_(S5A)_7_ strain in which the CTD contains seven Ser5 heptads. Apparently, there is a threshold level of Ser5 content that can sustain H3K4me and H3K36me3 modifications. By contrast, H2Bub1 was only reduced in the *rpb1-CTD-S5A-MCE1* strain, suggesting that this modification has a less stringent requirement for Rpb1 Ser5 content.

We observed a unique effect of Rpb1 Ser2 on H3K36 methylation, insofar as H3K36me3 levels, but not those of any other modification tested, were reduced in the *S2A* mutant strain (Figure 1A). Levels of H3K4me3 and H3K36me3 were also reduced, albeit less strongly, in *Y1F, T4A* and *S7A* mutant strains. The decrement in H3K4me3 levels observed in the *S7A* strain was smaller than that observed in the *S2A-S7A* double mutant, arguing that Ser2 and Ser7 have overlapping roles in positively regulating this modification. Thus, co-transcriptional methylation at H3K4 and H3K36 is influenced by multiple Rpb1 CTD phosphorylation sites. In contrast, global levels of H2Bub1 were not affected in the *S2A*, *Y1F*, *T4A*, *S7A* or *S2A-S7A* genetic backgrounds (Figure 1A).

We observed reduction of H2Bub1, H3K4me3 and H3K36me3 upon replacement of Ser7 with the phospho-mimetic glutamate residue (Figure 1A). The fact that a constitutive negative charge at this position generally impedes co-transcriptional modifications points to the dynamics of Rpb1 CTD phosphorylation as an important factor in their regulation.

As a further test of the role of the Rpb1 CTD in co-transcriptional histone ubiquitylation and methylation, we assessed the levels of these modifications in strains expressing Rpb1 subunits with CTDs of varying repeat lengths (5). We found that the levels of H2Bub1 and H3K36me3 were similar in strains carrying CTDs with 16, 11, 10 or 9 consensus repeats. H3K4me3 levels were reduced in lockstep with reduction of CTD repeat number, underscoring the sensitivity of H3K4me3 to Rpb1 CTD structure (Figure 1B).

### Overlapping and independent roles of the Rpb1 and Spt5 CTDs in promoting co-transcriptional methylation

**Figure 2.**
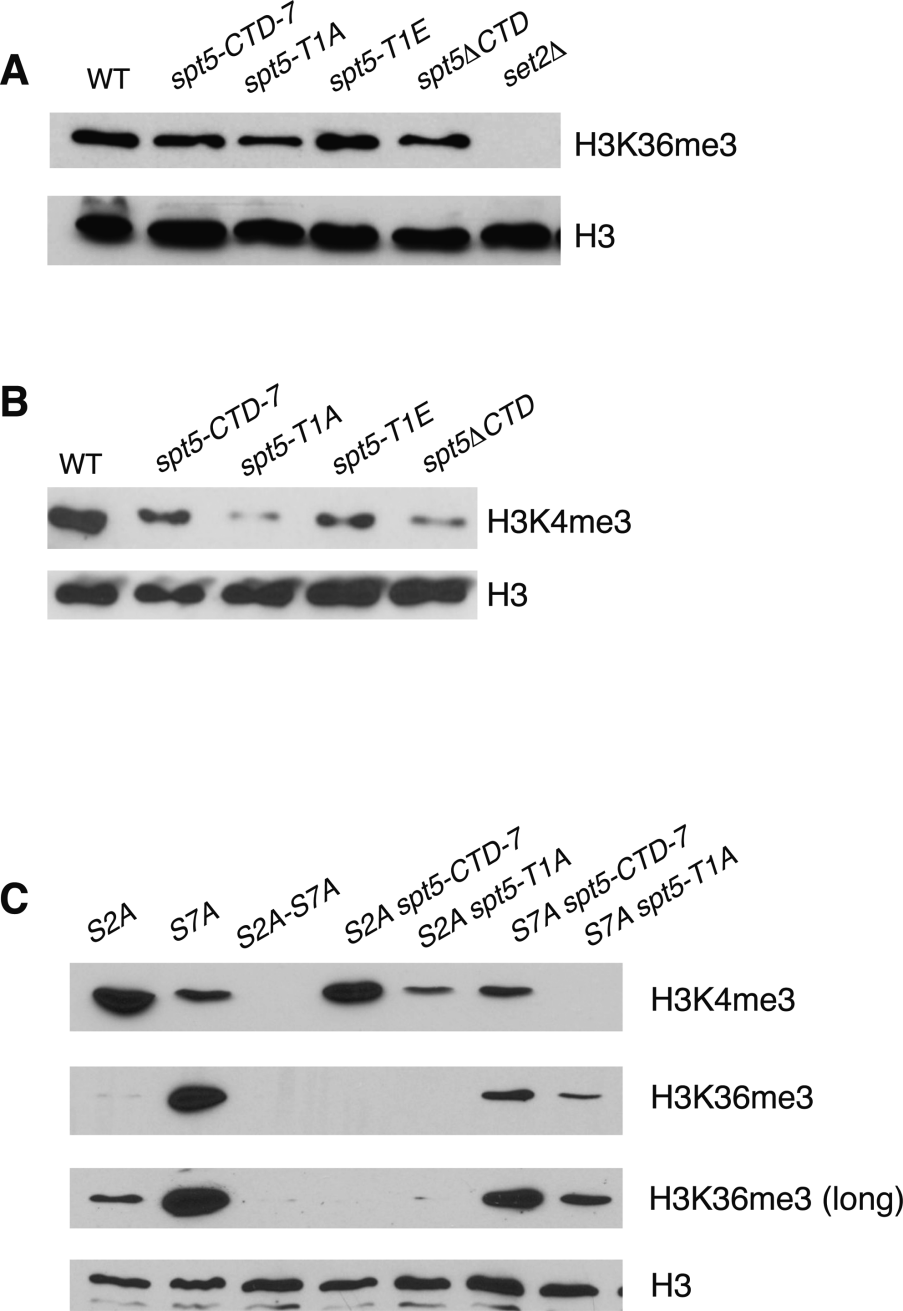
Role of the Spt5 CTD in formation of H3K36me3. **(A)** Whole-cell extracts derived from the indicated strains (top) were subjected to SDS-PAGE and immunoblotting with the indicated antibodies (right). ‘WT’ (wild-type) is described in Materials and Methods. The *spt5-CTD-7* strain harbors a truncated form of the CTD containing 7 consensus nonapeptide repeats that is functional for growth at all temperatures. The mutant strains harbor similar CTDs bearing the indicated mutations in all of the repeats or lack the CTD entirely (*spt5ΔCTD*). **(B)** As in (A). **(C)** As in (A) and Figure 1. Two exposures of the same H3K36me3 blot are shown.

Previous studies showed that H2Bub1 and H3K4me3 were both dependent upon phosphorylation of the Spt5 CTD (22). We thus tested whether H3K36me also required Spt5 CTD phosphorylation. We performed immunoblots on extracts prepared from strains expressing Spt5 with alanine or glutamate substitutions for Thr1 in all the repeats of a truncated (but functional) Spt5 CTD comprising 7 nonamer repeats (Spt5-CTD-7), or a version of Spt5 that lacks the entire CTD repeat array (ΔCTD). We observed little change in H3K36me3 in the *T1A* and Δ*CTD* strains, as compared to marked reductions in H3K4me3 (Figures 2A and B)(5). Thus, H3K36me3 is largely independent of Spt5 CTD phosphorylation.

To further assess the contributions of the Rpb1 and Spt5 CTDs to co-transcriptional histone modification mechanisms, we examined strains bearing mutations in both domains (5). The *spt5-T1A* mutation decreased H3K4me3 levels when combined with *rpb1-S2A* and led to an additive decrease in H3K4me3 in combination with *rpb1-S7A*, consistent with independent functions for the two CTDs in regulating H3K4me3 (Figure 2C). In the *rpb1-S7A* genetic background, H3K36me3 levels were reduced by the *spt5-CTD-7* allele, and the *spt5-T1A* allele elicited a greater reduction. In addition, the low levels of H3K36me3 detected in the *rpb1-S2A* genetic background were completely effaced by either *spt5* mutation (Figure 2C). These data demonstrate that H3K36me3 levels become sensitive to the length and phosphorylation state of the Spt5 CTD repeat array upon genetic alteration of the Rpb1 CTD, consistent with overlapping functions for the two CTDs.

It is possible that the observed effects of *rpb1* and *spt5* mutations on histone modifications are indirect results of impaired transcription elongation. We thus tested the growth of *rpb1* and *rpb1 spt5* double mutants in presence of the nucleotide biosynthesis inhibitor mycophenolic acid (MPA), sensitivity to which can often indicate a transcription elongation defect (27). Whereas growth of a hypomorphic *cdk9* mutant was markedly sensitive to MPA, no sensitivity was apparent for any of the *rpb1* or *spt5* mutants tested, suggesting that elongation is not generally impaired in these strains (Supplementary Figure S1).

### Global levels of Spt5 CTD phosphorylation are maintained in the absence of Rpb1 CTD Ser5 phosphorylation

**Figure 3.**
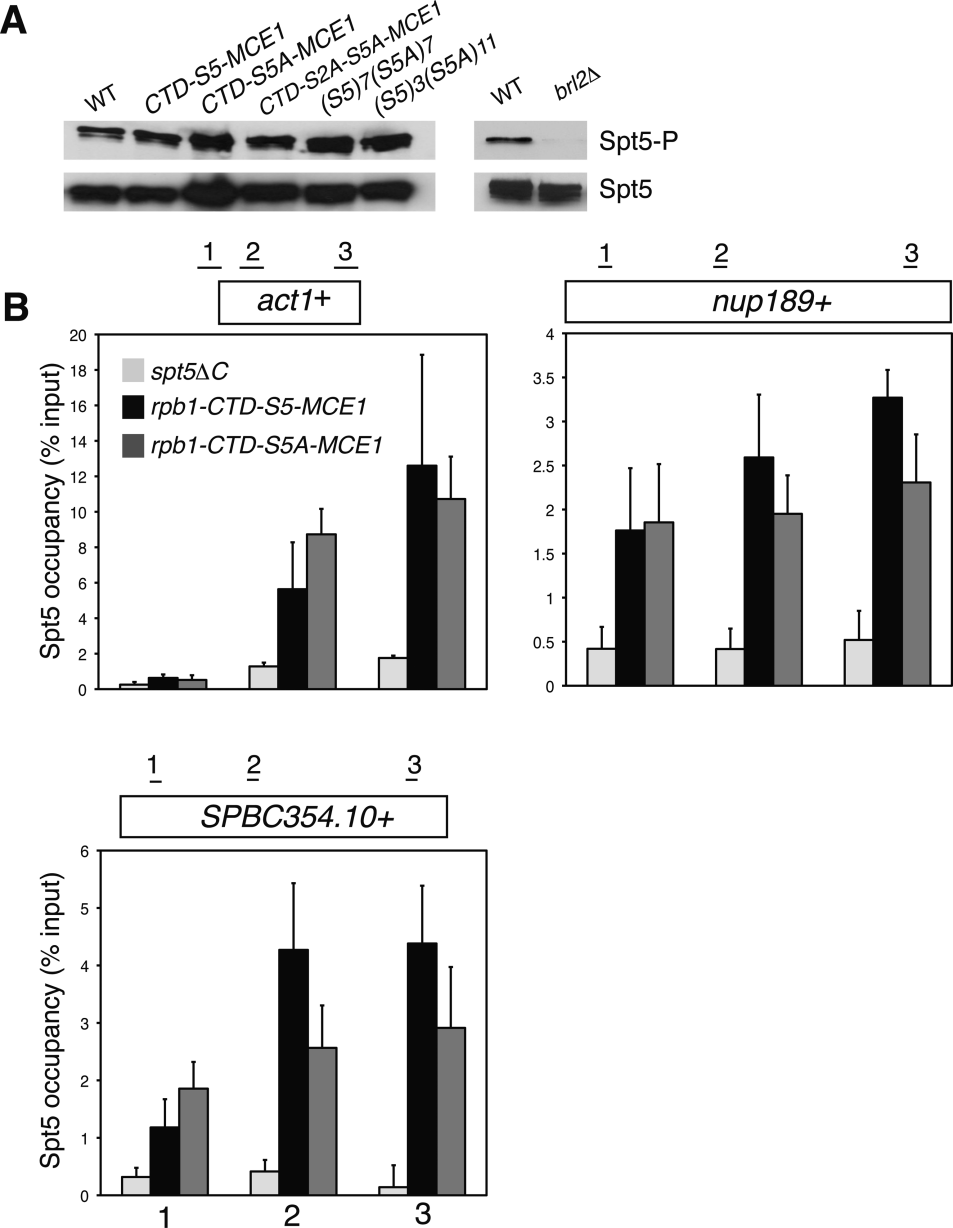
Loss of Rpb1 Ser5 does not impair Spt5 phosphorylation or its association with transcribed chromatin. **(A)** Whole-cell extracts derived from the indicated strains (top) were subjected to SDS-PAGE and immunoblotting with the indicated antibodies (right). ‘WT’ in the left panel is as in Figure 1; ‘WT’ in the right panel is as in Figure 2. **(B)** ChIP of Spt5 was carried out using a polyclonal antibody recognizing the Spt5 CTD in the indicated strains. A *spt5*Δ*C* strain lacking the entire CTD served as a negative control. ChIP signals were quantified by qPCR using primer pairs at the numbered locations across the *act1*^+^, *nup189*^+^ and *SPBC354.10*^+^ genes as shown and expressed as% of signal in the whole-cell extract (input). Error bars denote standard deviations from three independent experiments.

Since H2Bub1 and H3K4me depend on Spt5 CTD Thr1 (presumably via its phosphorylation), we tested whether the impact of Rpb1 Ser5 mutations on these modifications could reflect an effect of Rpb1 Ser5 on Spt5 T1-P. Immunoblotting using antibodies that recognize total Spt5 or Spt5 T1-P showed no effect of the Rpb1 S5A mutations on levels of Spt5 T1-P in extracts. As a control for these experiments we included extracts derived from a strain harboring a deletion of the *brl2^+^* gene, encoding a ubiquitin ligase for histone H2B. We observed decreased levels of Spt5 T1-P in the *brl2*Δ strain, in accord with the previously described positive feedback loop linking Spt5 T1-P and H2Bub1 (22)(Figure 3A).

Chromatin immunoprecipitation (ChIP) assays demonstrated that association of Spt5 with the *act1*^+^, *nup189*^+^ and *SPBC354.10*^+^ genes was similar in the *rpb1-CTD-S5-MCE1* and the *rpb1-CTD-S5A-MCE1* strains (Figure 3B). A control ChIP experiment using a strain lacking the Spt5 CTD confirmed the specificity of the ChIP signal. Therefore, neither phosphorylation of Spt5 nor its association with chromatin require Rpb1 Ser5. We conclude that Rpb1 Ser5 impacts co-transcriptional histone modifications independently of the Spt5 CTD.

### Dual roles of Lsk1/Cdk12-dependent Rpb1-S2P in regulating co-transcriptional histone methylation

**Figure 4.**
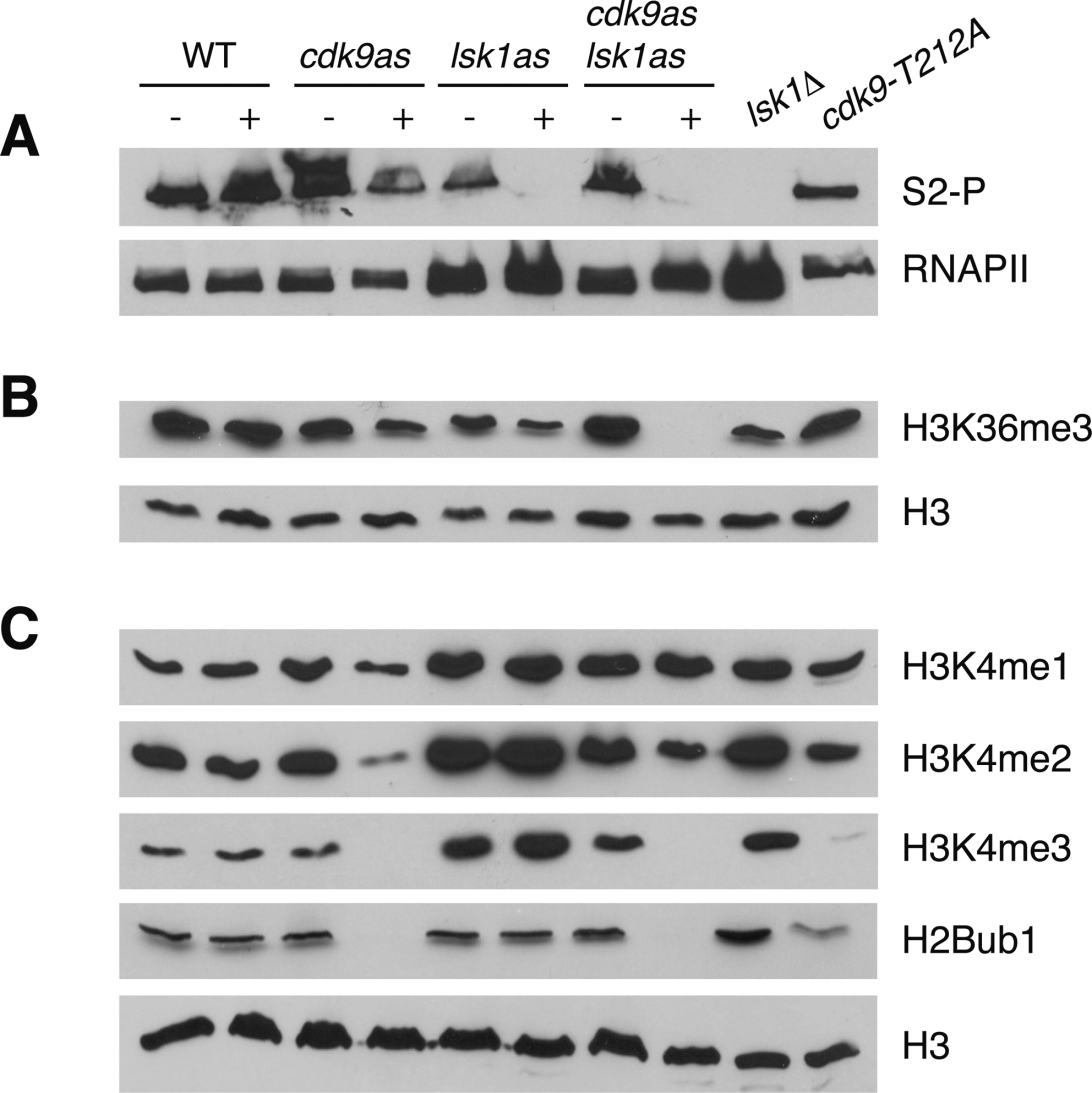
Positive and negative effects of Lsk1/Cdk12 on co-transcriptional histone modifications. **(A–C)** Whole-cell extracts derived from the indicated strains (top) were subjected to SDS-PAGE and immunoblotting with the indicated antibodies (right). For strains carrying analogue sensitive (as) alleles, cultures were treated with DMSO (−) or 20 μM 3-MB-PP1 (+) for 3 h prior to extract preparation.

To determine whether the effects for Rpb1 CTD Ser2 and Ser5 mutations on histone modification were attributable to the lack of phosphorylation, we inhibited Rpb1 CTD kinases implicated in phosphorylation of these sites. We employed strains harboring engineered ‘analogue sensitive’ (as) kinase variants that are inhibited by bulky ATP analogues. We focused first on kinases shown to target Rpb1 Ser2: Lsk1 (the *S. pombe* Cdk12 orthologue) and Cdk9. Inhibition of Lsk1^as^ via a 3-h treatment with 20 μM of the ATP analogue 3-MB-PP1 rendered the Rpb1 S2-P mark undetectable, as shown previously (Figure 4A) (48). Levels of H3K36me3 were reduced, similar to what we observed in the *rpb1-S2A* mutant strain (Figure 4B). A knockout of *lsk1*^+^ elicited effects on Rpb1 S2-P and H3K36me3 that were comparable to those produced by Lsk1^as^ inhibition, arguing that the effects of Lsk1 involve its kinase activity. These data also show that acute and chronic Lsk1 inactivation lead to similar effects on H3K36me3 levels (although inhibitor treatment for at least 2 h was required to observe the reduction in H3K36me3; see Supplementary Figure S2). We conclude that Lsk1 positively regulates H3K36me3 by phosphorylating Rpb1-Ser2.

Cdk9^as^ inhibition caused partial loss of H3K36me3 and Rpb1 S2-P (Figure 4A, B, and Supplementary Figure S2). The hypomorphic *cdk9-T212A* allele did not reproduce these effects, perhaps indicating that they require a greater reduction in Cdk9 activity (Figures 4A and B). As was observed for Lsk1^as^ inhibition, combined inhibition of Cdk9^as^ and Lsk1^as^ ablated Rpb1 S2-P (Figure 4A). Reduction of H3K36me3 was enhanced compared to that observed upon inhibition of either kinase individually (Figure 4B). This argues that steady-state levels of H3K36me are maintained by the activities of both Lsk1 and Cdk9, likely as a result of phosphorylation of Rpb1 Ser2 and Spt5 Thr1.

The *lsk1*Δ and *lsk1*^as^ mutations increased levels of H3K4me, arguing that Lsk1 negatively regulates methylation at H3K4 in a manner analogous to that found for *S. cerevisiae* Ctk1 (Figure 4C) (40,41). Loss of Ctk1 caused increases in H3K4me2 and H3K4me3 and a concomitant decrease in H3K4me1, whereas we found that all three H3K4 methylation states were similarly affected by *lsk1* mutations. The levels of H2Bub1 were not affected, indicating that regulation of H3K4me by Lsk1 does not involve the H2Bub1-H3K4me crosstalk pathway (57).

As expected based on our previous results (22), inhibition of Cdk9^as^ caused dramatic reduction in the levels of H2Bub1 and H3K4me3 (Figure 4C). Combined inhibition of Cdk9^as^ and Lsk1^as^ produced a similar effect, although Lsk1^as^ inhibition partially suppressed the effect of Cdk9 activity on H3K4me1 and H3K4me2. This indicates that the negative role of Lsk1 is downstream of Cdk9 activity.

Analogue-sensitive kinases are typically less active than their wild-type counterparts (even in the absence of analogue inhibitor) due to a reduction in ATP binding affinity. Thus, the fact that increased in H3K4me was apparent in the *lsk1^as^* strain in the absence of an analogue inhibitor could indicate that this effect is highly sensitive to reduction in Lsk1 activity.

**Figure 5.**
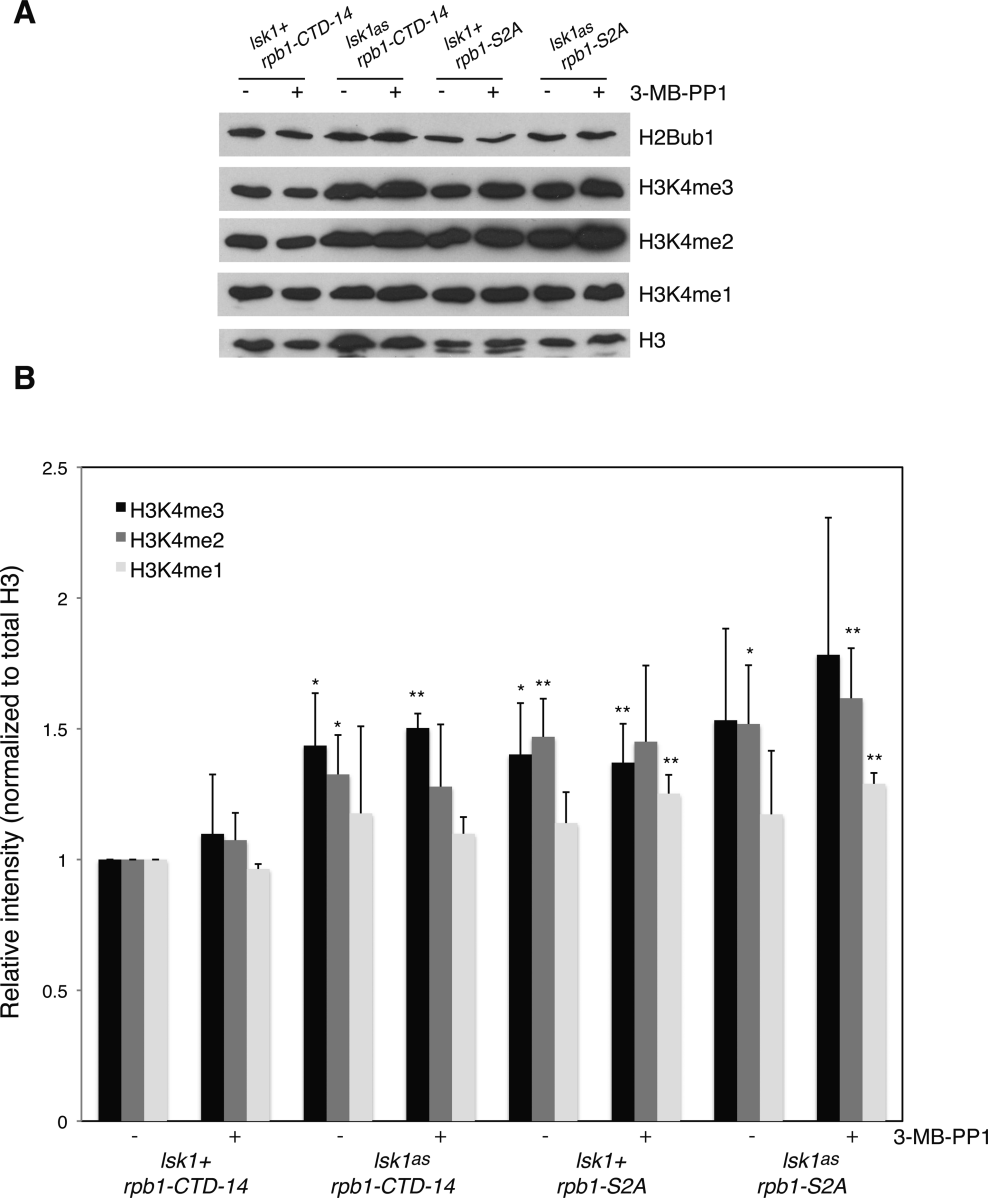
Lsk1/Cdk12 negatively regulates H3K4me via Rpb1 Ser2 phosphorylation. **(A)** Whole-cell extracts derived from the indicated strains (top) were subjected to SDS-PAGE and immunoblotting with the indicated antibodies (right). Cultures were treated with DMSO (−) or 20 μM 3-MB-PP1 (+) for 3 h prior to extract preparation. **(B)** Quantification of band intensities from three repeats of the experiment shown in (A). Intensities were determined using ImageJ software. In each experiment the H3K4me/H3 ratio in the DMSO-treated *rpb1-CTD-14* sample was set to 1. Significant increases over this value (as determined by student's t-test) are indicated (‘*’ denotes *P* < 0.1; ‘**’ denotes *P* < 0.05). Error bars denote standard deviations.

To determine whether regulation of H3K4me by Lsk1 occurred through phosphorylation of Rpb1-Ser2, we introduced the *lsk1^as^* allele into *rpb1-CTD-14* or *rpb1-S2A* strains. In the *rpb1-CTD-14* background, *lsk1^as^* caused a ∼50% increase in H3K4me3 and ∼30% increase in H3K4me2, as assessed by immunoblotting (Figure 5A and B). The *lsk1^as^* allele did not enhance H3K4me levels in the *rpb1-S2A* background, indicating that the effect of Lsk1 on H3K4me requires Rpb1 Ser2. Moreover, the S2A mutation by itself was sufficient to elicit increases in H3K4me2 and H3K4me3. Thus, Lsk1 negatively regulates H3K4me through phosphorylation of Rpb1-Ser2.

### The roles of Mcs6/Cdk7 and Rpb1 S5-P

**Figure 6.**
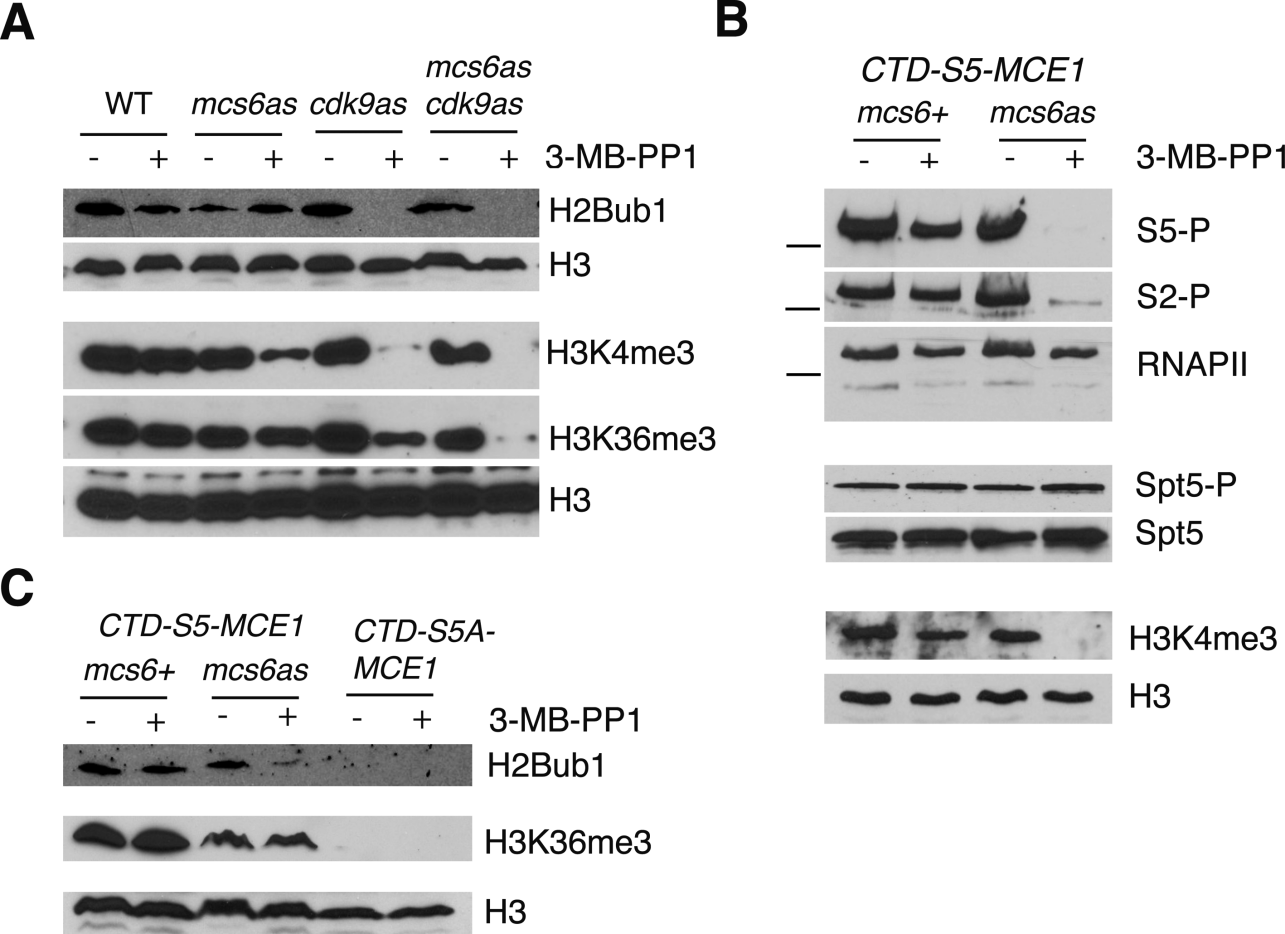
The role of Mcs6 activity in co-transcriptional histone modifications. **(A)** Strains carrying the indicated analogue sensitive (as) alleles were treated with DMSO (−) or 40 μM 3-MB-PP1 (+) for 3 h prior to extract preparation. Whole-cell extracts derived from each sample were analyzed by SDS-PAGE and immunoblotting with the indicated antibodies (right). **(B)** As in (A) for strains harboring *mcs6^+^* or *mcs6^as^* in combination with the *rpb1*-*CTD-S5-MCE1* allele. Dashes indicate the position of the 250 kD size marker. **(C)** As in (A) for strains harboring the indicated *mcs6* and *rpb1* alleles.

To examine the role of Rpb1 S5-P in co-transcriptional histone modifications we monitored modification levels upon inhibition of Mcs6^as^. Consistent with previous studies, treatment with 40 μM 3-MB-PP1 strongly reduced Rpb1 S5-P levels, but not those of Rpb1 S2-P, in the *mcs6^as^* strain (Supplementary Figure S3) (48,58). Global levels of H2Bub1 and H3K36me3 were unaffected under these conditions, but H3K4me3 levels were reduced (Figure 6A). This indicates that the sensitivity of H3K4me3 levels to alteration of Rpb1 Ser5 is likely due to the loss of Rpb1 S5-P. However, phosphorylation of Rpb1 Ser7, which also depends on Mcs6 activity, may contribute to this effect (58).

Combined inhibition of Mcs6^as^ and Cdk9^as^ decreased the levels of H3K36me3 more strongly than inhibition of either kinase individually (Figure 6A). However, this effect may be attributed to decreased Rpb1 S2-P and/or loss of Spt5 T1-P (Supplementary Figure S3 and (22)). Thus, our kinase inhibition experiments failed to link the requirement of Rpb1 Ser5 for co-transcriptional H2Bub1 and H3K36me to activity of Mcs6, the major kinase for Rpb1 Ser5 *in vivo*. We considered the possibility that residual Mcs6^as^ activity toward the Rpb1 CTD, maintained in the presence of inhibitory analogue, was sufficient for these histone modifications to occur. We thus introduced the *mcs6^as^* allele into a strain harboring the *rpb1-CTD-S5-MCE1* fusion. Rpb1 CTD function with respect to co-transcriptional histone modifications was partially compromised by its fusion to MCE1 (Figure 1A), but Cdk9-dependent phosphorylation of Spt5 was unaffected (Figure 3). We reasoned that *rpb1-CTD-S5-MCE1* might sensitize cells to the effects of Mcs6^as^ inhibition without affecting Spt5 T1-P. Levels of Rpb1 S5-P, Rpb1 S2-P and H3K4me3 were indeed more sensitive to inhibition of Mcs6^as^ in this background as compared to those in a strain with an intact Rpb1 CTD, although Spt5 T1-P levels were unaffected (Figure 6B and Supplementary Figure S3). The enhanced reduction of Rpb1 S5-P was also associated with a partial loss of H2Bub1 (as compared to that caused by *rpb1-CTD-S5A-MCE1*; Figure 6C). However, levels of H3K36me3 remained insensitive to Mcs6^as^ inhibition under these conditions (Figure 6C). H3K36me3 levels were similarly unaffected by Mcs6^as^ inhibition in a strain expressing Rpb1 with a truncated CTD lacking the MCE1 fusion (Supplementary Figure S4). These data suggest that the requirement of Rpb1 Ser5 for co-transcriptional H3K36me3 in *S. pombe* involves a mechanism that is independent of Mcs6/Cdk7, the major Rpb1 Ser5 kinase in this organism.

## DISCUSSION

**Figure 7.**
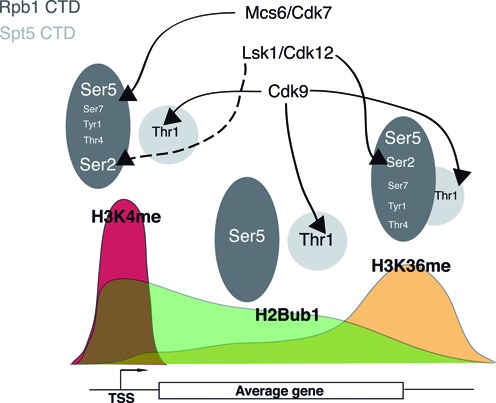
A proposed CTD ‘code’ for co-transcriptional histone modifications. Grey ovals depicting the Rpb1 and Spt5 CTDs are positioned above the typical histone modification landscape of a transcribed gene (see (11)). The ovals are labeled with the specific phospho-sites implicated in each of the indicated histone modifications and are depicted as independent or overlapping. The sizes of the labels denote their relative functional importance for each histone modification. Arrows connect CTD kinases to functionally validated target phospho-sites. Broken arrow indicates negative regulation of H3K4me by Lsk1/Cdk12-dependent Rpb1 Ser2 phosphorylation.

Our data provide the first comprehensive examination of the roles of known Rpb1 and Spt5 phosphorylation sites in co-transcriptional histone modifications (summarized in Figure 7). We observed the most dramatic reductions in steady-state levels of one or more modifications upon loss of Rpb1 Ser2, Rpb1 Ser5 and Spt5 Thr1, pointing to these as key CTD phosphorylation sites regulating co-transcriptional histone modification pathways. Nevertheless, our data indicate that the complete CTD ‘code’ directing these modifications is more complex and includes Rpb1 Tyr1, Rpb1 Thr4 and Rpb1 Ser7. Although previous experiments have demonstrated that phosphorylation of Rpb1 Ser2 and Rpb1 Ser5 does not require any of these sites, the *Y1F, T4A* and *S7A* mutations may have subtle effects on levels of Rpb1-S2P and/or Rpb1-S5P that contribute to their effects on histone modifications (47).

We found that Lsk1-dependent Rpb1 S2-P positively regulates H3K36me3, in agreement with previous work on Cdk12 orthologues in budding yeast and in nematodes (42,43). Our data also provide insights into the regulation of H3K4me by Lsk1 and other Cdk12 orthologues. In budding yeast, Ctk1 functions to prevent the aberrant accumulation of H3K4me3 at the 3′ ends of transcribed genes, and thus loss of Ctk1 causes a reduction in H3K4me1 and an increase in H3K4me3 (40,41). This function was shown to depend on Ctk1 kinase activity, although the relevant target was not identified (41). Our data demonstrate that the negative regulation of co-transcriptional H3K4me1, H3K4me2 and H3K4me3 by Lsk1 occurs via Rpb1 Ser2 phosphorylation. The *S2A* mutation also led to reduced H3K4me3 levels when combined with *S7A*, suggesting that unphosphorylated Rpb1 Ser2 makes a positive contribution to H3K4me.

In mammalian cells, Rpb1 S2-P is proposed to directly interact with WAC, a WW-domain protein and subunit of the RNF20/40 ubiquitin ligase complex for H2Bub1 (30). In addition, a version of Rpb1 lacking Ser2 in its CTD repeats cannot support normal levels of H2Bub1 (39). The fact that WAC is not conserved outside of metazoans could explain why we found Rpb1 Ser2 to be dispensable for H2Bub1 in *S. pombe*. Whether WAC may also interact with other CTD phosphorylation sites, such as Spt5 T1-P, has not been tested.

Examination of strains expressing mutant forms of both CTDs allowed us to assess their additive effects on co-transcriptional histone modifications. Consistent with the independent effects of Spt5 Thr1 and Rpb1 Ser7 on H3K4me3, removal of both of these sites resulted in further reduction of this modification. Loss of H3K4me3 caused by *spt5-T1A* is most likely secondary to reduction in H2Bub1 due to the H2Bub1-H3K4me crosstalk pathway (16,57). H3K4me3 levels were reduced in the *Y1F, S7A, T4A* and *S2A-S7A* genetic backgrounds, upon truncation of the Rpb1 CTD, and upon inhibition of Mcs6^as^. H2Bub1 levels were unaffected under these conditions. We think it likely that these effects reflect a direct interaction between the Set1C/COMPASS histone H3K4 methyltransferase complex and the phosphorylated Rpb1 CTD, an idea supported by previous reports (23,59,60). We thus envision Set1C/COMPASS as integrating independent signals from the Rpb1 and Spt5 CTDs during transcription.

Our data show that the Rpb1 and Spt5 CTDs have overlapping roles in the co-transcriptional formation of H3K36me. Although the ablation of Spt5 T1-P by itself has little effect on H3K36me3 levels, Spt5 T1-P becomes critical upon removal of Rpb1 Ser7. Whereas a primary role for the Rpb1 CTD in directing H3K36me is well established, a role for the Spt5 CTD has not been described previously. The apparent functional overlap could stem from impaired RNAPII elongation caused by defects in both CTD domains, although our MPA sensitivity experiments argued this was not the case in the mutant strains we employed.

H2Bub1 levels were insensitive to genetic alteration of most phospho-sites on the Rpb1 CTD, with the exception of Ser5. We were unable to assess functional overlap between these sites and Spt5 Thr1 as the levels of H2Bub1 in *spt5-T1A* mutants are already near the limits of detection in our immunoblots (22). However, Rpb1 Ser5 did not act through Spt5 Thr1, since levels of Spt5 T1-P were maintained and Spt5 crosslinking to several transcribed genes was not altered upon removal of Rpb1 Ser5. These data indicate that the deficit in H2Bub1 in S5A strains was not caused by a general impairment of Cdk9 activity toward Spt5 at sites of transcription. Our previous work also argues against a role for Rpb1 Ser5 in recruitment of the PAF complex or of the Rtf1 orthologue Prf1 to transcribed genes (28). Thus, Rpb1 Ser5 seems to define an alternate mechanism involved in co-transcriptional formation of H2Bub1.

The finding that H2Bub1 is independent of Mcs6 activity in *S. pombe* is in contrast to previous work on Cdk7 in mammalian cells and Kin28 in budding yeast. The requirement of Cdk7 activity for H2Bub1 was also uncovered by inhibition of an AS kinase expressed at physiologic levels, and can be explained by the CDK-activating function of this enzyme in mammalian cells, which is required for the activity of Cdk9 (31). In both fission and budding yeast, the CDK-activating function resides in an unrelated kinase (50,61). The role of Kin28 in H2Bub1 formation in budding yeast was ascertained using a temperature-sensitive *kin28* allele (*kin28ts-16-HA*), inactivation of which has been shown to disrupt the entire TFIIH complex at gene promoters (33,62). Thus, the effect of this allele on H2Bub1 may be unrelated to Kin28 activity per se.

How do we reconcile the requirement of Rpb1 Ser5 for H3K36me with the relative insensitivity of this mark to loss of Mcs6/Cdk7 activity? Phosphorylation of both Ser5 and Ser7 was eliminated and Ser2 phosphorylation was reduced slightly in the *rpb1-CTD-S5A-MCE1* strain (as assessed by immunoblotting of whole-cell extracts), raising the possibility that the *S5A* mutation exerts its effects by perturbing more than one mark (47). However, we found that phosphorylation of Rpb1 Ser5 and Ser2 are both decreased upon Mcs6^as^ inhibition in the *rpb1-CTD-S5-MCE1* background with minimal effects on H3K36me and H2Bub1. It is also possible that the effects of the *S5A* mutation on co-transcriptional histone modifications are an indirect consequence of chronic S5-P depletion, or are due to effects of *S5A* that are unrelated to Ser5 phosphorylation. Either of these possibilities would imply that H3K36me is in fact independent of Rpb1 S5-P per se, an idea that would be difficult to reconcile with the biochemical evidence linking the H3K36 methyltransferase Set2 to Rpb1 S5-P (24,25). We think it more likely that Rpb1 S5-P catalyzed by a kinase other than Mcs6/Cdk7 is necessary for co-transcriptional formation of H3K36me (and perhaps, to a lesser extent, H2Bub1). Cdk9 is the strongest candidate to fulfill this role for several reasons. First, genome-wide ChIP experiments in budding yeast demonstrate that the activities of Kin28 and Bur1 are both required for Rpb1 S5-P at promoter-distal locations within gene bodies (63). Second, although Cdk9 is thought to primarily target serine 2 on the Rpb1 CTD *in vivo*, experiments *in vitro* show that its preferred site on Rpb1 CTD-derived peptide substrates is in fact serine 5 (64). Finally, Cdk9 colocalizes with Rpb1 S5-P in ‘transcription factories’ visualized in live mammalian cells (65). Our data suggest a potential physiological role for Cdk9-dependent Rpb1 S5-P. An important goal of future work will be to uncover the mechanistic basis for the functional difference between Rpb1 S5-P catalyzed by Mcs6/Cdk7 and that catalyzed by Cdk9 or other kinases.

## Supplementary Material

SUPPLEMENTARY DATA
