# Supplementary material for: Functional interaction of Rpb1 and Spt5 C-terminal domains in co-transcriptional histone modification
Source: Nucleic Acids Res. 2015 Aug 14;43(20):9766–75. doi: 10.1093/nar/gkv837 (PMC4787787; doi:10.1093/nar/gkv837)
Supplement: SUPPLEMENTARY DATA [file supp_43_20_9766__index.html]

Functional interaction of Rpb1 and Spt5 C-terminal domains in co-transcriptional histone modification — Functional interaction of Rpb1 and Spt5 C-terminal domains in co-transcriptional histone modification — SUPPLEMENTARY DATA 

# Functional interaction of Rpb1 and Spt5 C-terminal domains in co-transcriptional histone modification

## SUPPLEMENTARY DATA

- SUPPLEMENTARY DATA
