# Supplementary material for: Functional interaction of Rpb1 and Spt5 C-terminal domains in co-transcriptional histone modification
Source: Nucleic Acids Res. 2015 Aug 14;43(20):9766–75. doi: 10.1093/nar/gkv837 (PMC4787787; doi:10.1093/nar/gkv837)
Supplement: SUPPLEMENTARY DATA [file supp_gkv837_nar-00858-x-2015-File009.pdf]

Functional interaction of Rpb1 and Spt5 C-terminal domains in co-transcriptional histone modification

Jean Mbogning[1], Viviane Pagé[1], Jillian Burston[1], Emily Schwenger[1], Robert P. Fisher[2], Beate Schwer[3], Stewart Shuman[4], and Jason C. Tanny[1]*

Supplementary Data

Figures S1-S4

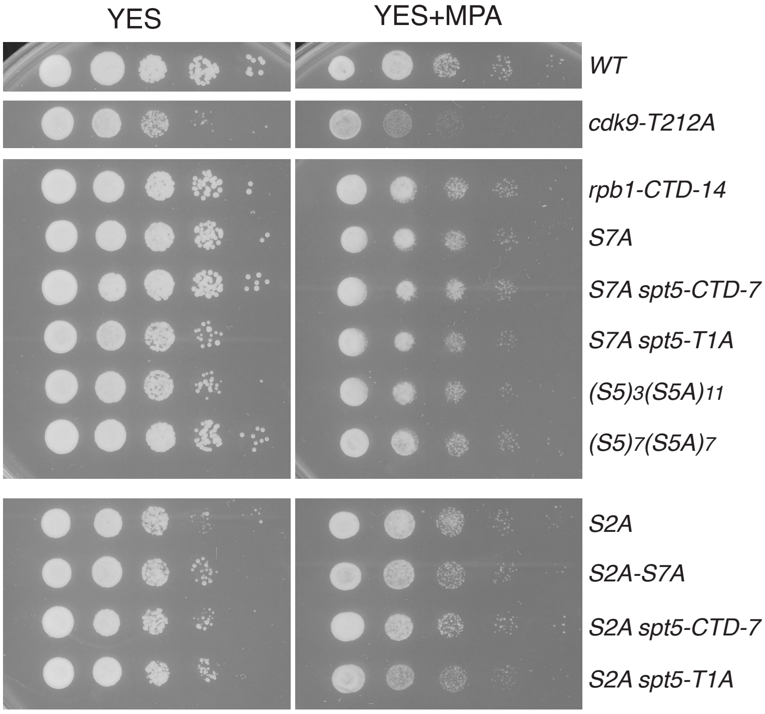

Figure S1. Growth of *rpb1* and *spt5* mutant strains is not sensitive to mycophenolic acid (MPA). The indicated strains were grown to mid-log phase in liquid culture and 5-fold serial dilutions were spotted on rich media (YES) plates containing DMSO (left) or 25 μg/mL MPA (right). Plates were incubated at 30°C for 2-3 days.

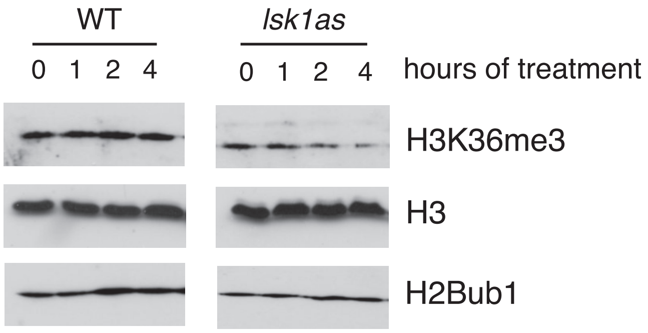

Figure S2. Time course of analogue inhibitor treatment in wild-type and *lsk1*^{as} strains. Wild-type (WT) and *lsk1*^{as} strains were treated with DMSO (-) or 20 μM 3-MB-PP1 (+) for the indicated times prior to extract preparation. Whole-cell extracts derived from each sample were analyzed by SDS-PAGE and immunoblotting with the indicated antibodies (right).

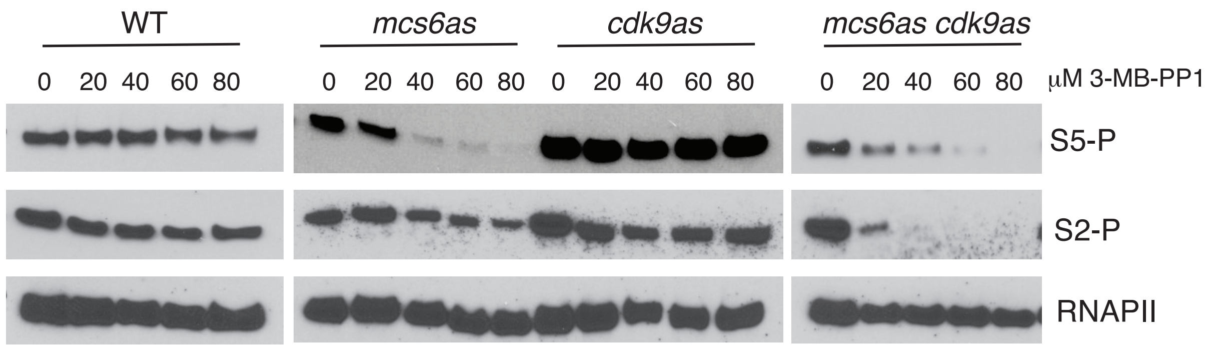

Figure S3. Roles of Mcs6 and Cdk9 in Rpb1 CTD phosphorylation. Strains carrying the indicated analogue sensitive alleles (top) were treated with DMSO (-) or the indicated concentrations of 3-MB-PP1 for three hours. Whole-cell extracts derived from each sample were analyzed by SDS-PAGE and immunoblotting with the indicated antibodies (right).

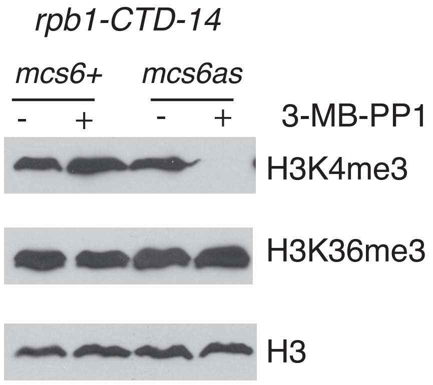

Figure S4. H3K36me3 is independent of Mcs6 activity in the context of a truncated Rpb1 CTD. Strains carrying the indicated *mcs6* alleles in combination with a truncated *rpb1* allele (14 CTD repeats) were treated with DMSO (-) or 40 μM 3-MB-PP1 (+) for three hours prior to extract preparation. Whole-cell extracts derived from each sample were analyzed by SDS-PAGE and immunoblotting with the indicated antibodies (right).
